# Mineralogical and Microstructural Characteristics of Two Dental Pulp Capping Materials

**DOI:** 10.3390/ma12111772

**Published:** 2019-05-31

**Authors:** Georgeta Voicu, Andreea C. Didilescu, Andrei B. Stoian, Cristina Dumitriu, Maria Greabu, Mihai Andrei

**Affiliations:** 1 Department of Biomaterials and Medical Devices, Faculty of Medical Engineering, University Politehnica of Bucharest, 1-7 Polizu, 011061 Bucharest, Romania; getav2001@yahoo.co.uk; 2 Division of Embryology, Faculty of Dental Medicine, Carol Davila University of Medicine and Pharmacy, 8 Eroii Sanitari Boulevard, 050474 Bucharest, Romania; dr.andrei.mihai@gmail.com; 3 Department of General Chemistry, Faculty of Applied Chemistry and Materials Science, University Politehnica of Bucharest, University Politehnica of Bucharest, 1-7 Polizu, 011061 Bucharest, Romania; andreibstoian@yahoo.com (A.B.S.); cristina.dumitriu@upb.ro (C.D.); 4 Division of Biochemistry, Carol Davila University of Medicine and Pharmacy, 8 Eroii Sanitari Boulevard, 050474 Bucharest, Romania; maria.greabu@umfcd.ro

**Keywords:** tricalcium silicate, hydration, mineralization, apatite, MTA, TheraCal

## Abstract

This paper aims to investigate the composition, surface, and microstructural characteristics, and bioactivity of two commercially available pulp capping materials known as TheraCal LC and BIO MTA+. The materials were prepared as cylindrical samples and assessed by X-ray diffraction (XRD) and complex thermal analysis for mineralogical characterization, and by scanning electron microscopy (SEM) coupled with energy dispersive of X-ray (EDX), Fourier-Transformed Infrared Spectroscopy (FT-IR), and atomic force microscopy (AFM) for microstructural and surface characteristics. The in vitro bioactivity was highlighted by surface mineralization throughout SEM coupled with EDX and FT-IR analysis. XRD analysis performed on both materials showed calcium silicate phases and different radiopacifying compounds. AFM measurements indicated a smoother and more homogenous surface with a lower average roughness for TheraCal LC due to the resin matrix from its composition. FT-IR analysis displayed bands for several compounds in both materials. Both materials exhibited bioactive properties showing surface mineralization after being immersed in solution similar to the human physiological environment. However, the MTA cement showed a better mineralization due to the anhydrous and hydrated phases.

## 1. Introduction

Pulp capping is a clinical procedure involving the placement of a biomaterial, which aims to maintain dental pulp vitality by stimulating the formation of tertiary dentin as a hard-tissue barrier protector. Over time, a number of materials have been developed to maintain pulp vitality as pulp capping agents. Calcium hydroxide has been a gold standard for a long time, but its drawbacks such as lack of adhesion to dentin or other filling materials, lack of proper sealing, and the presence of micro-porosity leading to the formation of tunnel defects that favored bacterial infiltration at the pulp level [1], have led to the production of more intricate materials. It has been recently demonstrated that mineral trioxide aggregate (MTA) has a superior effect on hard-tissue barrier formation when compared with calcium hydroxide [2].

In 1990, MTA was developed and initially approached as an endodontic material for the repair of root defects [3] or for the induction of apical closure of immature teeth [4,5]. MTA is a hydraulic cement, and a tricalcium silicate-based material. The MTA composition has predominantly Portland type I cement, which has the main crystalline solid phases: alite (tricalcium silicate, 3CaO·SiO_2_, C_3_S), belite (dicalcium silicate, 2CaO·SiO_2_, C_2_S), cellite (tetracalcium aluminoferrite, 4CaO·Al_2_O_3_·Fe_2_O_3_, C_4_AF) and tricalcium alluminate (3CaO·Al_2_O_3_, C_3_A), with the following main oxide components: CaO, SiO_2_, Al_2_O_3_, and Fe_2_O_3_ [6,7,8]. Besides this, in the MTA’s composition there are Ba and Sr oxides with opacifying roles [9]. Following the setting reaction, called hydration, by mixing the powder with aqueous solution, compounds such as calcium hydroxide and calcium silicate hydrate are formed [7,8,9,10]. The MTA setting reaction, which can be carried out in wet environments, was one of the great advantages besides excellent biocompatibility, alkaline pH, good marginal closure, and a much lower inflammatory response than calcium hydroxide in direct pulp capping [11,12]. However, MTA has a number of disadvantages when used as a pulp capping material. The prolonged setting reaction time requires more clinical interventions, difficult maneuverability, and a delay of the final restoration [13,14], compatibility issues when associated with other filling materials, affecting their setting reaction [15,16], as well as color stability over time [17,18].

In 2011, TheraCal, a light cured resin-modified tricalcium silicate, was introduced as a pulp capping agent. TheraCal is based on tricalcium silicate particles dispersed in a hydrophilic monomer to allow calcium release. TheraCal is composed of main mineralogical phases of Portland cement (e.g., calcium silicates), thickening agents, resin matrix, and barium sulphate as a radiopaque agent. The easy handling and application of the TheraCal, as well as the short time of setting reaction, the possibility of use as a base or liner material under resin or amalgam fillings [19], and the lack of necessity for dentine conditioning prior to application, raise the main advantages.

Both materials are bioactive and capable of inducing remineralization by their ability to form apatite using calcium silicates and/or calcium aluminates [10,20]. This results in dentin bridge formation [21,22]. Although TheraCal exhibited some favorable results when compared to those shown by MTA, this material has been overlooked [23].

The outcomes of this study provide new insights into the field of calcium silicate-based dental materials. The specific aims were to in vitro assess and compare: (i) the chemical composition, (ii) the surface and microstructural characteristics, and (iii) biomineralization (bioactivity) of two commercially available pulp capping materials: TheraCal LC and BIO MTA+, with different setting reaction mechanisms.

## 2. Materials and Methods

The two materials included in the study were TheraCal LC (Bisco, Schaumburg, IL, USA) and BIO MTA+ (Cerkamed, Stalowa Wola, Poland). Both materials were prepared as cylindrical specimens. The samples were prepared following the manufacturer’s indications for use. TheraCal was applied from the provided syringe in layers of 1 mm and was light cured for 20 s for each increment. BIO MTA+ cement was prepared by mixing the content of the silicate powder with one-two drops of aqueous liquid, until the compound reached a standard consistency. The hardening took place to 37 °C, R.H. = 100% (R.H.—relative humidity), for 3 days. Seven samples were obtained for each material, among which 4 were used for biomaterial characterization, and 3 for bioactivity investigation.

The in vitro bioactivity of both materials was assessed on hardened TheraCal samples by polymerization and hardened BIO MTA+ samples for 3 days at 37 °C, R.H. = 100%. For this, the hardened samples were soaked in simulated body fluid (SBF) (the specimen area to SBF volume ratio was 0.1 cm^−1^). The samples were stored for 28 days in water bath at 37 °C and then removed, gently washed with distilled water in order to remove all the soluble salts precipitated from SBF and dried at 60 °C for 24 h. Three samples for each material were used for bioactivity investigation. The SBF had a similar composition with human blood plasma, according to Kokubo’s solution [24].

Samples were analyzed for the following characteristics: (i) mineralogical, by X-ray diffraction (XRD), complex thermal analysis (DTA-TG), (ii) microstructural and surface properties, by scanning electron microscopy (SEM) coupled with energy dispersive of X-ray (EDX), Fourier-transformed infrared spectroscopy (FT-IR), and atomic force microscopy (AFM). In addition, in vitro bioactivity was qualitatively assessed by SEM coupled with EDX and FT-IR, which highlights the surface mineralization.

Thermogravimetric Analysis (TGA/DrTGA) and Differential Thermal Analysis (DTA) of tricalcium silicate were performed on TheraCal samples, in order to establish the material’s mineralogical composition. Complex thermal analysis was performed using a DT GTA-50H derivatograph (Shimadzu, Kyoto, Japan), between 20 and 1000 °C, with a heating rate of 10 °C/min, in an oxidizing atmosphere.

XRD was used to obtain information on the degree of crystallinity of the samples. It was performed with an XRD 6000 diffractometer (Shimadzu, Kyoto, Japan) with filtered radiation—Ni CuKα (λ = 1.5406 Å) at a speed scanning at 2°/min, in the 2*θ* range of 5–75°.

SEM coupled with EDX was performed with a Quanta INSPECT F electronic scanning microscope (FEI Company, Hillsboro, OR, USA) with a resolution of 1.2 nm, equipped with an EDX detector with a MnKalpha resolution of 1.33 eV. The samples were gold-coated.

Atomic Force Microscopy (AFM) imaging was performed in Contact Mode using an A100-SGS microscope (APE Research, Trieste, Italy) in order to investigate the topography of the samples. The raw data were processed with Gwyddion software. The tip used with the AFM was a type HQ:CSC17/Al BS from MikroMasch (Wetzlar, Germany) made from n-type silicon coated with aluminum with a total tip height of 12–18 µm, a typical radius of the tip was 8 nm, and a total full tip cone angle was 40°. The cantilever had the following dimensions: length 450 µm, width 50 µm, thickness 2 µm, a typical force constant of 0.18 N/m, and a typical resonance frequency of 13 kHz.

An ATR/FT-IR Spectrum 100 (Perkin Elmer, Waltham, MA, USA) in absorption mode was used for spectral scanning in the region of 4000 to 600 cm^−1^. Presented spectra were recorded using the average of four scans with 4 cm^−1^ resolution. The ATR/FT-IR control recorded and processed spectra data where performed using Spectrum software.

## 3. Results

### 3.1. Complex Thermal Analysis

**Figure 1:**
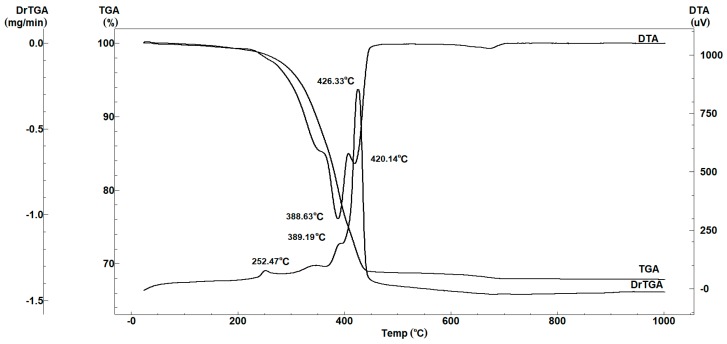
Thermogravimetric and differential thermal analysis results of the resin modified tricalcium silicate.

Complex thermal analysis was performed on hardened TheraCal as shown in Figure 1, in order to be show evidence of the nature of component phases. Three exothermic effects, one after another, with mass loss, can be observed on the DTA thermogram, with a stronger effect at 426 °C. The mass loss can be attributed to the thermal decomposition and burning of the organic component of the resin modified sample. The endothermic effect is very low at above 700 °C and it is accompanied by mass loss on the thermogravimetric curve (about 0.6%). This process can be attributed to the decomposition process for a low-grade structured carbonate. Based on the results of thermal analysis for identification of the crystalline mineral phase of the hardened material, it was thermally treated at 500 °C for 2 h, after which X-ray diffraction analysis was performed.

### 3.2. X-ray Diffraction Analysis

**Figure 2:**
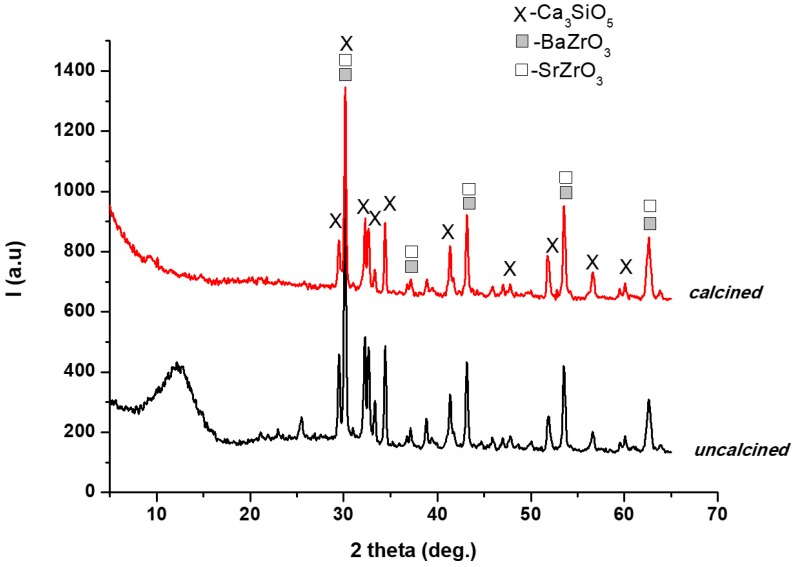
XRD patterns for calcined and uncalcined hardened TheraCal.

The results of XRD analysis on calcined and uncalcined hardened TheraCal are shown in Figure 2. The XRD patterns suggested that the mineral phase was mainly formed from tricalcium silicate (Ca_3_SiO_5_, PCDFWIN [042-0551]) and two rich phases of Ba and Sr (BaZrO_3_-PCDFWIN [006-0399], SrZrO_3_-PCDFWIN [023-0561]). The halo from the small angles range (5°–20°) for the uncalcined sample was due to the presence of the organic phase.

**Figure 3:**
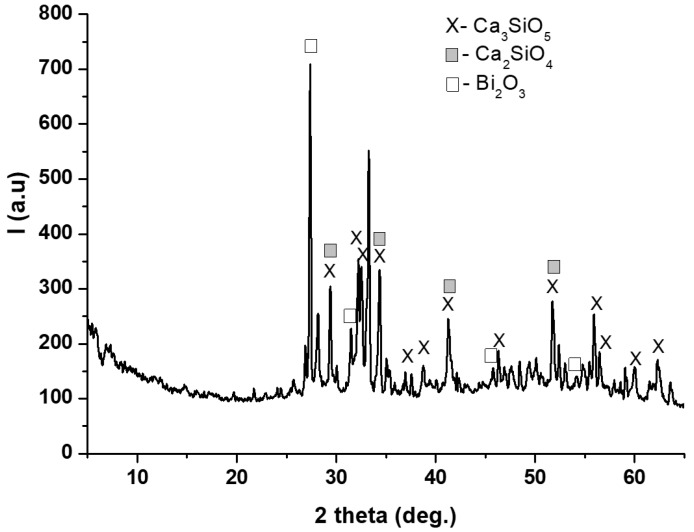
XRD patterns for anhydrous BIO MTA+ powder.

The XRD pattern of anhydrous BIO MTA+ powder showed the main mineralogical phases: tricalcium silicate (Ca_3_SiO_5_, PCDFWIN [042-0551]), dicalcium silicate (Ca_2_SiO_4_, PCDFWIN [036-0642]) and bismuth oxide (Bi_2_O_3_- PCDFWIN [071-0466]) (Figure 3).

### 3.3. Scanning Electron Microsopy Coupled with EDX

**Figure 4:**
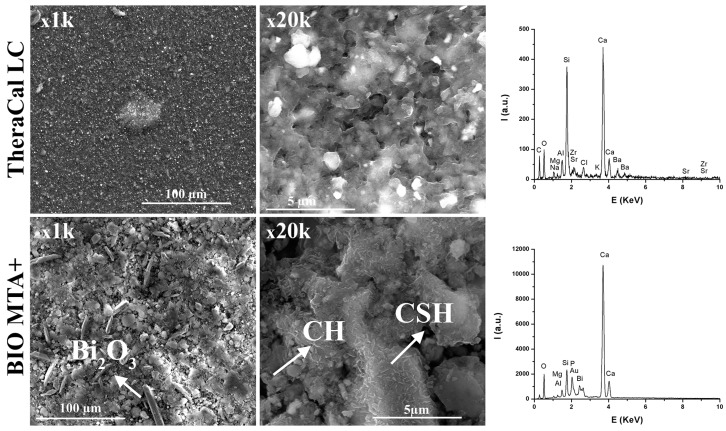
Scanning electron micrographs at different magnifications and EDX spectra of TheraCal and BIO MTA+ surfaces at three days.

Scanning electron microscopy was initially performed on both hardened material samples (Figure 4). The hardened TheraCal sample displayed mineral particles evenly distributed in the organic matrix, characterized by the presence of a micro-porosity, most likely due to the air contained during the material application (Figure 4). The EDX spectrum was in correlation with XRD results, which show the presence of characteristic elements of the mineral phase.

**Figure 5:**
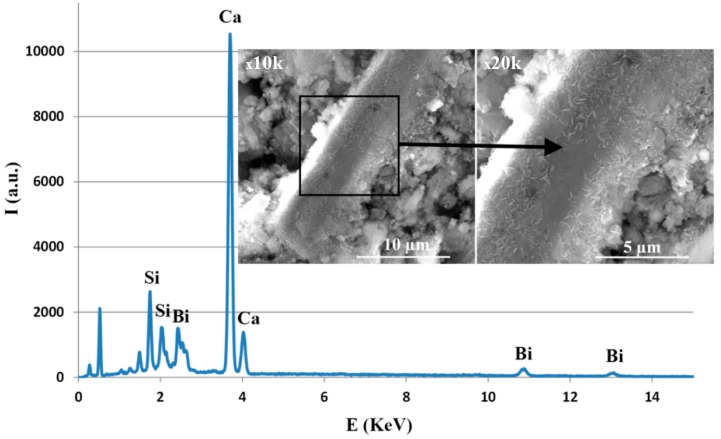
SEM image for the hardened MTA at three days, 37 °C, R.H. (relative humidity) = 100%, with detail on the Bi_2_O_3_ particle and characteristic EDX spectra.

For the BIO MTA+ sample hardened at three days, at 37 °C, R.H. = 100%, a dense microstructure with a porosity under 10 µm could be observed (Figure 4). The EDX spectrum was in good concordance with XRD results. The presence of P was most likely due to a phosphate phase that was below the detection limit of the diffractometer or had a low degree of crystallinity. The presence of Bi_2_O_3_, in the form of rods, and the main hydration phases—calcium silicate hydrates (CSH) in the form needle-like/folded sheets and calcium hydroxide (CH) in the form of hexagonal plates—were also visible. We also observed that hydration phases, especially CSH, were deposited on the Bi_2_O_3_ surface (Figure 5), which may suggest that Bi_2_O_3_ may play a role on the crystallization substrate. The EDX spectrum demonstrated the deposition of CSH on the Bi_2_O_3_ particles’ surface (Figure 5, square area).

### 3.4. Atomic Force Microscopy

**Figure 6:**
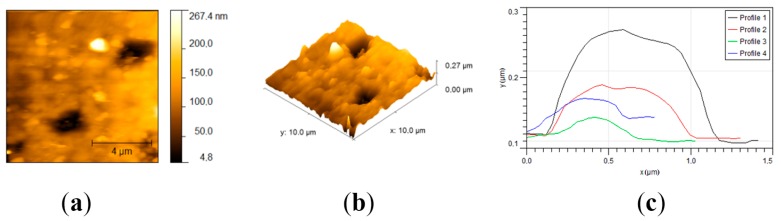
AFM micrographs and statistical data of the tricalcium silicate surface. (**a**) Top view; (**b**) 3D view; (**c**) crystals profiles.

The topography of both sample surfaces was determined throughout AFM measurements. For the TheraCal sample (Figure 6), AFM measurements showed a relatively smooth surface with an average roughness (Ra) of 29.9 nm and a root mean square roughness (Rms) of 38.3 nm. Skewness was used to measure the profile symmetry to the mean line. For the analyzed sample, a skewness value of 0.0407 hinted that the height distribution was symmetrical. The Kurtosis value of 1.11 indicated that the surface was relatively flat. The topography of the sample revealed that some crystals were protruding from the surface, with lengths varying from 0.3 to 1.2 μm, heights between 30 and 160 nm, and almost circular gaps with diameters around 1.6 μm and depths of around 40 nm.

**Figure 7:**
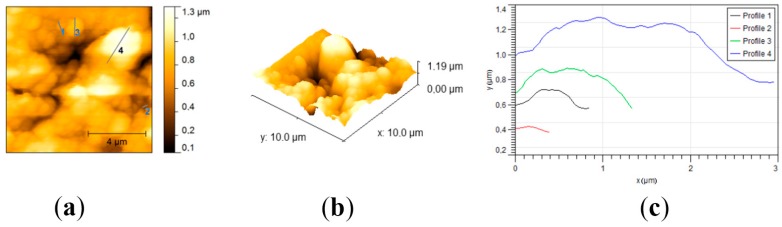
AFM micrographs and statistical data of the MTA surface. (**a**) Top view; (**b**) 3D view; (**c**) crystals profiles.

Regarding BIO MTA+, the sample had a rough surface with an average roughness (Ra) of 0.173 µm and a root mean square roughness (Rms) of 0.215 µm (Figure 7). For the analyzed sample, a skewness value of −0.508 hinted that the height distribution was shifted to higher values than the median. This means that there were more protrusions on the sample. The Kurtosis value of −0.173 indicated that the height distribution was skewed above the mean plane. The topography of the sample revealed that the surface was covered with bumps. The bumps were formed by aggregates of crystals with diameters of around 300 nm, but the biggest found aggregates reached 3 µm.

### 3.5. Bioactivity

**Figure 8:**
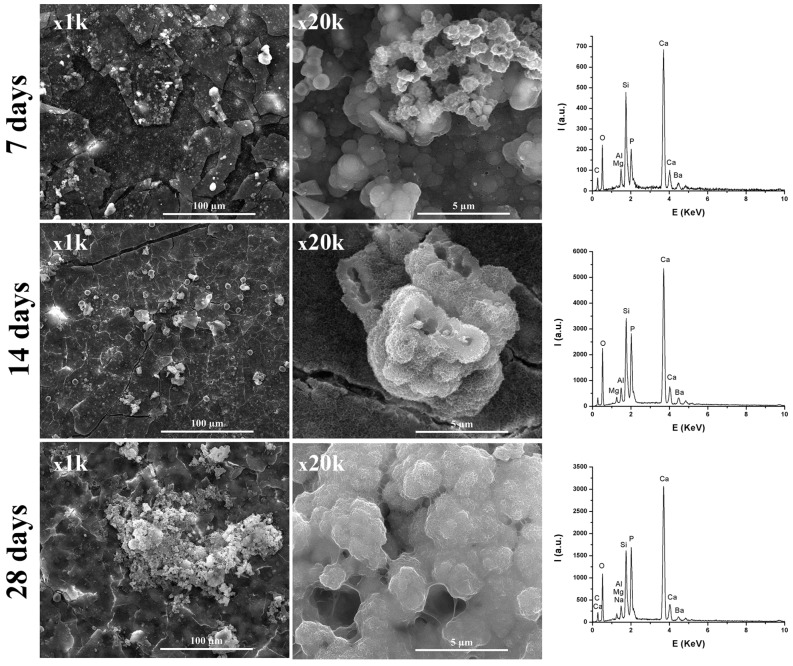
Scanning electron micrographs at different magnifications and elemental analysis of the TheraCal surface at 7, 14, and 28 days, which show mineralization.

**Figure 9:**
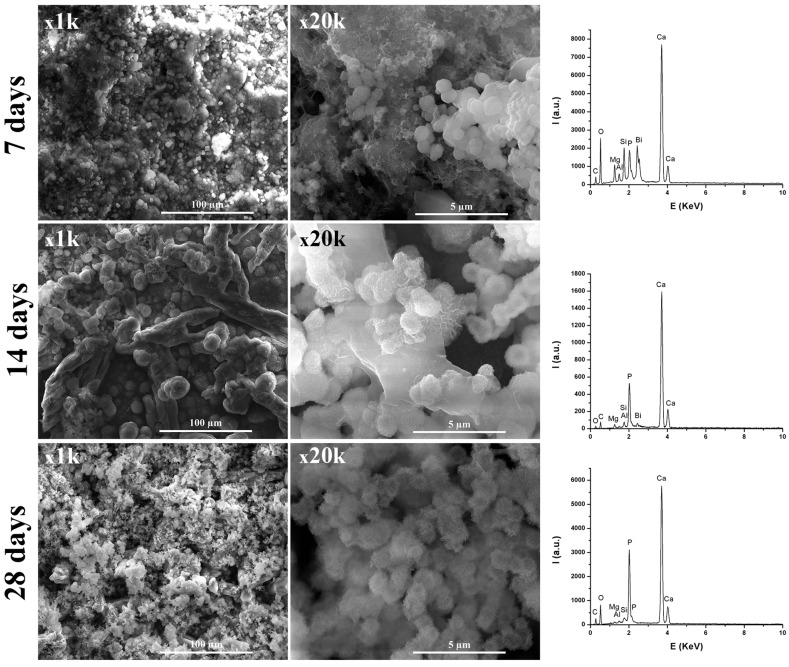
Scanning electron micrographs at different magnifications and elemental analysis of the BIO MTA+ surface showing mineralization at 7, 14, and 28 days.

From Figure 8 and Figure 9, it can be observed that, by immersing in SBF solution, at 7, 14, and 28 days, the materials surface was mineralized, due to the formation of apatite phases arranged in plaques that formed spheres. The formation of these apatite phases was sustained by the EDX spectra, where an important intensity of the P element was observed. Additionally, the presence of Si from CSH resulted by hydration-hydrolysis of calcium silicates.

Regarding the TheraCal sample, the presence of Si along with Ca was observed, which suggests formation of hydrosilicates in the presence of SBF. In the case of the BIO MTA+ sample immersed in SBF solution, the intensity of Si decreased with the immersion time in SBF, which suggests deposition on CSH surfaces of apatite phases. Additionally, from Figure 8 and Figure 9, it was noticeable that BIO-MTA+ mineralization was much better than TheraCal’s, most likely due to the slower diffusion of Ca ions from TheraCal in solution, in order to form hydroxyapatite.

### 3.6. Fourier Transformed Infrared Spectroscopy

**Figure 10:**
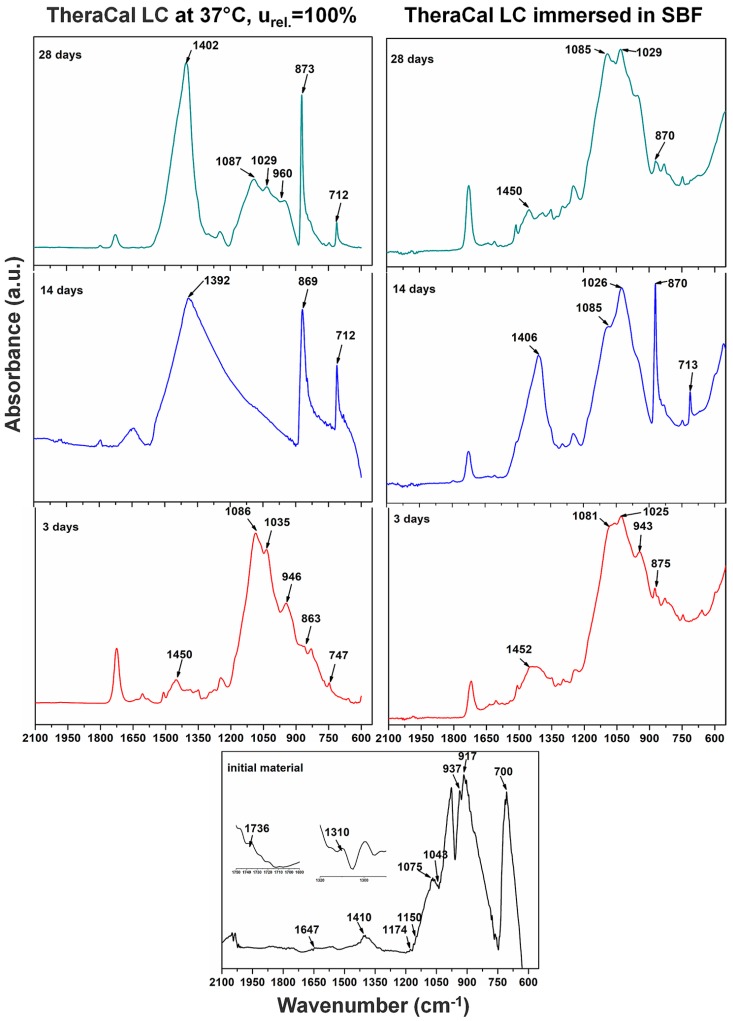
FT-IR spectra for TheraCal, and immersion in SBF (simulated body fluid).

**Figure 11:**
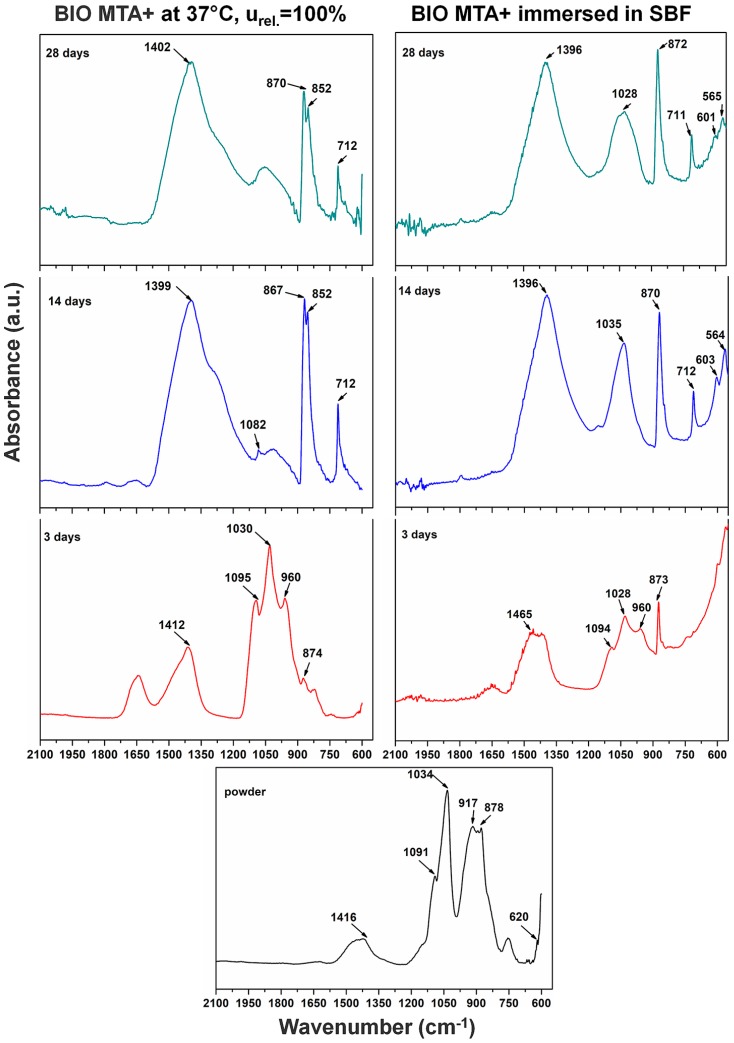
FT-IR spectra for BIO MTA+ during the setting reaction and immersion in SBF.

Materials were analyzed using FT-IR to determine changes during the initial setting reaction and after. This was conducted at 3, 14, and 28 days of immersion in SBF (Figure 10 and Figure 11).

The initial TheraCal spectrum showed a CO_3_^2−^ band at 700 cm^−1^ and a band at 917 cm^−1^ assigned to SiO_4_^4−^ stretching for the alite crystal phase. A characteristic bending for alite (Ca_3_SiO_5_) could be seen at 937 cm^−1^. Bands of PO_4_^3−^ groups were visible at 1043 and 1075 cm^−1^ and a band for asymmetric CO_3_^2−^ stretching at 1410 cm^−1^. These bands were attributed to apatite (HA-Ca_10_(PO_4_)_6_(OH)_2_) and calcite, which could not be detected by XRD analyses. There were also bands attributed to the polymer components: 1150 and 1310 cm^−1^ for SO_2_, 1174 cm^−1^ for COO– band, C=O at 1736 cm^−1^, and benzene C=C at 1647 cm^−1^.

After three days from the setting reaction, TheraCal showed a shift for the CO_3_^2−^ band corresponding from 1410 to 1450 cm^−1^. The CO_3_^2−^ band was also confirmed by the appearance of a band at 863 cm^−1^. The prominent band at 1086 cm^−1^ corresponded to CO_3_^2−^. Another corresponding band to CO_3_^2−^ was shifted from 700 to 747 cm^−1^. At 946 cm^−1^, there was a band assigned to CSH and, at 1035 cm^−1^, there was a band for PO_4_^3−^ asymmetric stretching from apatite. After 14 days, TheraCal spectrum showed some changes, with CO_3_^2−^ band corresponding to a crystal phase of calcite being shifted to a lower frequency at 1392 cm^−1^. The CO_3_^2−^ band was also visible at 863 cm^−1^. Calcium carbonate was highlighted by a band at 712 cm^−1^. TheraCal spectra at 28 days showed an intense band at 1402 cm^−1^, which was attributed to CO_3_^2−^ stretching from calcite and hydroxycarbonate apatite (HCA). Bands at 1029 and 960 cm^−1^ were attributed to PO_4_^3−^ (from apatite). In addition, a band at 960 cm^−1^ was assigned for CSH. After immersion in SBF, a band at 1029 to 1025 cm^−1^ was attributed to PO_4_^3−^ from apatite. CSH was highlighted at 946 cm^−1^. The bands at 1081 cm^−1^ and 875 cm^−1^ showed the presence of CO_3_^2−^ from calcite, and 1450, 1406, 1085, and 870 cm^−1^ from HCA.

BIO MTA+ powder spectrum showed some differences when compared to the TheraCal’s initial material spectrum. The SO_4_^2−^ band from anhydrite or gypsum was present at 620 cm^−1^. The band at 917 cm^−1^ was assigned for SiO_4_^4−^ stretching from alite (Ca_3_SiO_5_) and bands at 878-873 cm^−1^ showed the presence of SiO_4_^4−^, which were assigned for belite. By hydration, the band at 960 cm^−1^ could be attributed for CSH.

By immersion in SBF, a high band at 1034 cm^−1^ was assigned to PO_4_^3−^ from apatite. The CO_3_^2−^ stretching from calcite (CaCO_3_) and HCA was visible at 1416 cm^−1^. For samples immersed in SBF at 3 and 14 days, bands at 1465, 1400, and 1081 cm^−1^ were assigned to CO_3_^2−^ corresponding to the formation of calcite and/or HCA. Bands at 1200 cm^−1^ and 960 cm^−1^ (3 days) as well as 600 cm^−1^ and 560 cm^−1^ (14 days), were assigned to PO_4_^3−^, which indicates symmetric stretching from apatite (HA). After 14 days, bands at ~1400, 1090–870 cm^−1^ were visible. The band at ~710 cm^−1^ was assigned for CO_3_^2−^ bending from calcite. In the recorded spectra after 28 days of immersion, bands were similar with those after 14 days of immersion in SBF. In the 28 days spectra, compared to the one at 14 days, the band at ~1090 cm^−1^ was absent.

## 4. Discussion

The aim of the study was to compare, in terms of composition, bioactivity, surface, and microstructural characteristics, two biomaterials that perform the same clinical function (dentin bridge formation), but are chemically binder systems with different setting reaction mechanisms: TheraCal LC, a light cured tricalcium silicate, and BIO MTA+, a mineral trioxide aggregate cement. Both materials exhibit bioactive properties interacting with the aqueous medium and forming apatite phases, which contribute to surface mineralization.

Characterization is an important step in the materials research because of changes that may occur during processing and testing [25]. In this respect, X-ray diffraction used for the compositional and crystal phase characterization and performed on TheraCal samples indicated the presence of the following mineral phases: Ca_3_SiO_5_, BaZrO_3_, and SrZrO_5_, which are the main constituents of Portland cement [26]. In addition, SEM images showed uniform distribution of these phases in the polymeric matrix and EDX spectrum demonstrated the presence of barium and strontium as radiopacifying elements. The diffractogram performed on BIO MTA+ anhydrous powder indicated the presence of calcium silicates as major phases—Ca_3_SiO_5_, Ca_2_SiO_4_, and Bi_2_O_3_ [7,8,27,28,29]. The addition of Bi_2_O_3_ is required, radiopacity being one of the basic requirements of a dental material [30], observing that hydro compounds resulting from the setting reaction are deposited on its surface.

The results of FT-IR analysis for TheraCal presented similar bands for alite (Ca_3_SiO_5_), and apatite (HA-C_10_(PO_4_)_6_(OH)_2_) [31], CO_3_^2−^, SiO_4_^4−^ [32], CSH [33], and calcium carbonate [34], which is similar to literature data, and, after the setting reaction, bands for CSH could be identified. By immersion in SBF, it was observed that characteristic bands of the phosphate groups increased, due to mineralization of the hardened material surface. FT-IR analysis performed on the anhydrous BIO MTA+ powder showed bands for CO_3_^2−^ and alite, which is similar to those obtained by Abu Zeid al. [34] and Gandolfi et al. [32]. By hydration at 37 °C, R.H. = 100%, calcium silicates were converted into calcium silicates hydrates, with their presence being demonstrated by scanning electron microscopy images through the form of needle-like/foils [35] and FT-IR spectra [32].

The results of AFM measurements indicated that the TheraCal sample had an overall smoother surface than the BIO MTA+ sample. The smaller mineral crystals were dispersed slightly well in the resin matrix of TheraCal, which makes the surface more homogeneous. The circular gaps found in the film could have been formed by micro air bubbles trapped in the film. This aspect was in concordance with the SEM micrographs, which also indicated the presence of surface microporosity. Since there was no resin involved in the fabrication of BIO MTA+, the mineral aggregates varied greatly in size for this sample and were less well dispersed, which give the surface higher roughness values. The surface of the sample had no visible baseline and the tops of the aggregates were found to have relatively sharp edges. The increased surface roughness of BIO MTA+ and lack of resin matrix may lead to better deposition of hydroxyapatite after 28 days of immersion in the SBF solution. Surface roughness, which is an important material characteristic, mirrors not only the surface texture, but also the vertical deviations from an ideal flat surface [36]. It plays an important role in bioactivity and cellular adherence. A rougher surface provides a better biocompatibility by promoting cellular attachment, proliferation, and growth, with a more favorable biological response [37,38]. Nevertheless, undue surface roughness associated with calcium depletion may negatively affect the strength and sealing properties of materials [39].

The in vitro behavior of the two materials was studied using FT-IR spectroscopy and SEM imaging at 3, 14, and 28 days by their immersion in SBF solution. In both materials, the formation of apatite phases was identified as spheres made of aggregates of plaques. These aspects were confirmed by EDX spectra associated with SEM measurements. The typical SEM imaging aspect of mineralization was given by the presence of spherical agglomerates formed by plaques of apatite. CSH needle-like formations have also been observed. The same aspects were also mentioned by Abu Zeid et al. [34] in the SEM characterization of two different commercial MTA cements. In case of TheraCal, the presence of calcium phosphate on the surface was highlighted, as indicated by the presence of the P element in EDX spectra, and PO_4_^3−^ in FT-IR spectra.

Study limitations include lack of investigations on mechanical behavior and material stability in high humidity environments. Regarding the TheraCal’s setting reaction, it ensures that, the first phase hardening, is due to the polymerization processes in the presence of UV light. Hardening can continue in aqueous medium, with the formation of calcium silicate hydrates. This provides good mechanical behavior. However, further investigations are needed.

## 5. Conclusions

Although both materials contain silicates, BIO MTA+ displayed more mineral phases. TheraCal LC revealed a smoother surface after the setting reaction, due to the resin in its composition, while BIO MTA+’s surface was rougher due to its higher mineralization. Both materials have bioactive properties, which develop apatite on their surfaces, after immersion in a solution similar to the human physiological environment. This mineralization was better for BIO MTA+ material, with both the anhydrous and hydrated phases constituting apatite deposition surfaces. From these perspectives, MTA seems to be a better choice than TheraCal for dentin regeneration.

## References

[1] Aguilar P., Linsuwanont P. (2011). Vital pulp therapy in vital permanent teeth with cariously exposed pulp: A systematic review. J. Endod..

[2] Didilescu A.C., Cristache C.M., Andrei M., Voicu G., Perlea P. (2018). The effect of dental pulp-capping materials on hard-tissue barrier formation: A systematic review and meta-analysis. J. Am. Dent. Assoc..

[3] Song M., Yu B., Kim S., Hayashi M., Smith C., Sohn S., Kim E., Lim J., Stevenson R.G., Kim R.H. (2017). Clinical and Molecular Perspectives of Reparative Dentin Formation: Lessons Learned from Pulp-Capping Materials and the Emerging Roles of Calcium. Dent. Clin. North. Am..

[4] Camilleri J., Pitt Ford T.R. (2006). Mineral trioxide aggregate: A review of the constituents and biological properties of the material. Int. Endod. J..

[5] Felippe W.T., Felippe M.C., Rocha M.J. (2006). The effect of mineral trioxide aggregate on the apexification and periapical healing of teeth with incomplete root formation. Int. Endod. J..

[6] Kaur M., Singh H., Dhillon J.S., Batra M., Saini M. (2017). MTA versus Biodentine: Review of Literature with a Comparative Analysis. J. Clinical Diagnostic Res. JCDR.

[7] Voicu G., Bădănoiu A.I., Andronescu E., Chifiriuc C.M. (2013). Synthesis, characterization and bioevaluation of partially stabilized cements for medical applications. Centr. Eur. J. Chem..

[8] Voicu G., Popa A.M., Badanoiu A.I., Iordache F. (2016). Influence of Thermal Treatment Conditions on the Properties of Dental Silicate Cements. Molecules.

[9] Comin-Chiaramonti L., Cavalleri G., Sbaizero O., Comin-Chiaramonti P. (2009). Crystallochemical comparison between Portland cements and mineral trioxide aggregate (MTA). J. Appl. Biomater. Biomech..

[10] Camilleri J. (2007). Hydration mechanisms of mineral trioxide aggregate. Int. Endod. J..

[11] Nair P.N., Duncan H.F., Pitt Ford T.R., Luder H.U. (2008). Histological, ultrastructural and quantitative investigations on the response of healthy human pulps to experimental capping with mineral trioxide aggregate: A randomized controlled trial. Int. Endod. J..

[12] Bogen G., Kim J.S., Bakland L.K. (2008). Direct pulp capping with mineral trioxide aggregate: An observational study. J. Am. Dent. Assoc..

[13] Chng H.K., Islam I., Yap A.U., Tong Y.W., Koh E.T. (2005). Properties of a new root-end filling material. J. Endod..

[14] Palma P.J., Marques J.A., Falacho R.I., Vinagre A., Santos J.M., Ramos J.C. (2018). Does Delayed Restoration Improve Shear Bond Strength of Different Restorative Protocols to Calcium Silicate-Based Cements?. Materials (Basel).

[15] Komabayashi T., Zhu Q., Eberhart R., Imai Y. (2016). Current status of direct pulp-capping materials for permanent teeth. Dent. Mater. J..

[16] Camilleri J. (2011). Scanning electron microscopic evaluation of the material interface of adjacent layers of dental materials. Dent. Mater..

[17] Camilleri J. (2014). Color stability of white mineral trioxide aggregate in contact with hypochlorite solution. J. Endod..

[18] Palma P.J., Marques J.A., Falacho R.I., Correia E., Vinagre A., Santos J.M., Ramos J.C. (2019). Six-Month Color Stability Assessment of Two Calcium Silicate-Based Cements Used in Regenerative Endodontic Procedures. J. Funct. Biomater..

[19] Qureshi A., Soujanya E., Nandakumar P. (2014). Recent advances in pulp capping materials: An overview. J. Clinical Diagnostic Res. JCDR.

[20] Yamamoto S., Han L., Noiri Y., Okiji T. (2017). Evaluation of the Ca ion release, pH and surface apatite formation of a prototype tricalcium silicate cement. Int. Endod. J..

[21] Maroto M., Barberia E., Planells P., Garcia Godoy F. (2005). Dentin bridge formation after mineral trioxide aggregate (MTA) pulpotomies in primary teeth. Am. J. Dent..

[22] Gandolfi M.G., Siboni F., Prati C. (2012). Chemical-physical properties of TheraCal, a novel light-curable MTA-like material for pulp capping. Int. Endod. J..

[23] Emara R., Elhennawy K., Schwendicke F. (2018). Effects of calcium silicate cements on dental pulp cells: A systematic review. J. Dent..

[24] Kokubo T., Takadama H. (2006). How useful is SBF in predicting in vivo bone bioactivity?. Biomaterials.

[25] Meraji N., Camilleri J. (2017). Bonding over Dentin Replacement Materials. J. Endod..

[26] Camilleri J. (2014). Hydration characteristics of Biodentine and Theracal used as pulp capping materials. Dent. Mater..

[27] Basturk F.B., Nekoofar M.H., Gunday M., Dummer P.M.H. (2018). X-ray diffraction analysis of MTA mixed and placed with various techniques. Clin. Oral. Investig..

[28] Guven Y., Tuna E.B., Dincol M.E., Aktoren O. (2014). X-ray diffraction analysis of MTA-Plus, MTA-Angelus and DiaRoot BioAggregate. Eur. J. Dent..

[29] Li Q., Coleman N.J. (2015). The hydration chemistry of ProRoot MTA. Dent. Mater. J..

[30] Grazziotin-Soares R., Nekoofar M.H., Davies T.E., Bafail A., Alhaddar E., Hubler R., Busato A.L., Dummer P.M. (2014). Effect of bismuth oxide on white mineral trioxide aggregate: Chemical characterization and physical properties. Int. Endod. J..

[31] Gong V., Franca R. (2017). Nanoscale chemical surface characterization of four different types of dental pulp-capping materials. J. Dent..

[32] Gandolfi M.G., Taddei P., Tinti A., Prati C. (2010). Apatite-forming ability (bioactivity) of ProRoot MTA. Int. Endod. J..

[33] Lee Y.L., Wang W.H., Lin F.H., Lin C.P. (2017). Hydration behaviors of calcium silicate-based biomaterials. J. Formos. Med. Assoc..

[34] Abu Zeid S.T., Alamoudi N.M., Khafagi M.G., Abou Neel E.A. (2017). Chemistry and Bioactivity of NeoMTA Plus™ versus MTA Angelus^®^ Root Repair Materials. J. Spectroscopy.

[35] Camilleri J., Montesin F.E., Brady K., Sweeney R., Curtis R.V., Ford T.R. (2005). The constitution of mineral trioxide aggregate. Dent. Mater..

[36] Aksel H., Kucukkaya Eren S., Askerbeyli Ors S., Karaismailoglu E. (2018). Surface and vertical dimensional changes of mineral trioxide aggregate and biodentine in different environmental conditions. J. Appl. Oral. Sci..

[37] Collado-Gonzalez M., Garcia-Bernal D., Onate-Sanchez R.E., Ortolani-Seltenerich P.S., Alvarez-Muro T., Lozano A., Forner L., Llena C., Moraleda J.M., Rodriguez-Lozano F.J. (2017). Cytotoxicity and bioactivity of various pulpotomy materials on stem cells from human exfoliated primary teeth. Int. Endod. J..

[38] Shi W., Mozumder M.S., Zhang H., Zhu J., Perinpanayagam H. (2012). MTA-enriched nanocomposite TiO(2)-polymeric powder coatings support human mesenchymal cell attachment and growth. Biomed. Mater..

[39] Smith J.B., Loushine R.J., Weller R.N., Rueggeberg F.A., Whitford G.M., Pashley D.H., Tay F.R. (2007). Metrologic evaluation of the surface of white MTA after the use of two endodontic irrigants. J. Endod..

